# Modulatory role of *Faecalibacterium* on insulin resistance and coagulation in patients with post-viral long haulers depending on adiposity

**DOI:** 10.1016/j.isci.2024.110450

**Published:** 2024-07-06

**Authors:** Amanda Cuevas-Sierra, Lourdes Chero-Sandoval, Andrea Higuera-Gómez, J. Antonio Vargas, María Martínez-Urbistondo, Raquel Castejón, J. Alfredo Martínez

**Affiliations:** 1 Precision Nutrition and Cardiometabolic Health, IMDEA-Food Institute (Madrid Institute for Advanced Studies), Campus of International Excellence (CEI) UAM+CSIC, 28049 Madrid, Spain; 2 Department of Endocrinology and Nutrition of the University Clinical Hospital, University of Valladolid, 47002 Valladolid, Spain; 3 Internal Medicine Service of Puerta de Hierro Majadahonda University Hospital, 2822 Madrid, Spain; 4 Centro de Medicina y Endocrinología, Universidad de Valladolid, Valladolid, Spain; 5 Centro de Investigación Biomédica en Red de la Fisiopatología de la Obesidad y Nutrición (CIBERobn), Instituto de Salud Carlos III, Madrid, Spain

**Keywords:** Human metabolism, Microbiology, Virology

## Abstract

Patients with Post-viral long hauler encompass lasting symptoms and comorbid complexities, often exacerbated in individuals with excessive body weight. The aim was to study gut microbiota in 130 patients with post-viral long hauler stratified by body mass index (BMI) and the relationship between inflammation and microbiota. Significant higher values were found for anthropometric variables and markers of glucose and dyslipidemia in individuals with higher BMI, as well as elevated levels of C-reactive protein, fibrinogen, IL-6, uric acid, and D-dimer. An interactive association showed an interplay between *Faecalibacterium*, D-dimer levels, and insulin resistance. This investigation showed that anthropometric, biochemical, and inflammatory variables were impaired in patients with post-viral long haulers with higher BMI. In addition, gut microbiota differences were found between groups and a modification effect on *Faecalibacterium* abundance regarding insulin resistance and D-dimer. These findings suggest that considering adiposity and gut microbiota structure and composition may improve personalized clinical interventions in patients with chronic inflammation.

## Highlights

• Long-haulers with higher BMI showed elevated glucose, HOMA-IR, and insulin levels
• Long-haulers with higher BMI exhibited higher CRP, fibrinogen, D-dimer, and uric acid
• Gut microbiota varied by BMI, with *Faecalibacterium* linked to insulin resistance

## Graphical abstract

**Figure:**
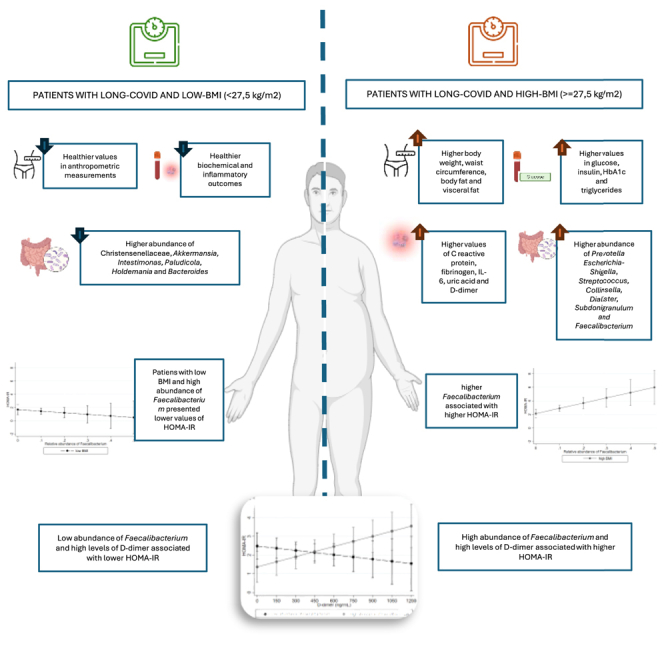

## Introduction

The viral infection of coronavirus SARS-Cov2 showcases a diverse spectrum of clinical presentations,^1^ but after the onset of the pandemic, where it was rapidly recognized that people with comorbidities and in particular metabolic diseases have a higher risk of developing severe symptoms and increased mortality.^2^ The WHO defines long-COVID (LC) as the manifestation of symptoms from a "probable or confirmed" COVID-19 infection that start approximately 3 months after the onset of the acute infection and last for more than 2 months.^3^ Several comorbidities, such as diabetes, obesity, hypertension, and cardiovascular diseases, are linked to poorer prognoses and could complicate the clearance of the virus, being these group of patients more often affected by LC and to experience more long-term consequences than general population.^4^ In this line, a body mass index (BMI) exceeding 40 kg/m^2^ emerged as the second most influential independent predictor of hospitalization in patients with SARS-CoV-2.^5^ Obesity is characterized by the excessive accumulation of adipose tissue, intimately associated with chronic low-grade inflammation.^6^ In this context, adipose tissue, functioning as a dynamic endocrine organ, releases a spectrum of proinflammatory cytokines, chemokines, and adipokines that orchestrate immune responses and metabolic processes.^7^ Moreover, ACE2, produced by adipocytes, may facilitate viral entry into adipose tissue, potentially serving as a viral reservoir.^8^ Dysregulated adipose-derived signaling molecules contribute to the establishment of a proinflammatory milieu, typified by elevated levels of tumor necrosis factor-alpha (TNF-α), interleukin-6 (IL-6), and C-reactive protein (CRP), among others.^9^ In fact, following viral infection and because of the inflammatory state, some groups of patients can present hyperglycemia, insulin resistance and insulin secretory deficits from impaired β cells, either due to direct viral damage or indirect effects.^10^ Beyond direct cellular infection, several other mechanisms exist which may explain the pathophysiology leading to COVID-19 multiorgan systemic disorder, including complications such as endothelial injury, immune system dysregulation, and hypercoagulability often leading to thrombosis.^11^

An immune system dysregulation has been suggested due to the finding of autoreactive T cells in autopsies of deceased individuals infected with COVID-19, likely due to mechanisms such as those in autoimmune disease.^12^ Additionally, studies have shown that chronic viral infections are associated with coagulation and thrombosis.^13^ Retrospective studies have identified common thrombotic biomarkers in patients with COVID-19, which help elucidate the underlying mechanisms of COVID-19-related thrombosis.^14^ In addition to disease severity, the occurrence of impaired blood variables and the elevated levels of coagulation markers are important determinants of prognosis in patients with COVID-19.^15^

Recent attention has underscored the pivotal role of gut microbiota in regulating immune responses and inflammatory processes, particularly in the context of excess of body weight. Emerging evidence indicates that alterations in gut microbiota composition and function contribute to the pathogenesis and progression of inflammatory disorders by modulating immune regulation and tissue homeostasis.^16^ Dysbiosis, an imbalance in microbial communities, has been correlated with metabolic disturbances and adipose tissue inflammation, further underscoring the intricate interplay between gut microbiota, chronic inflammation, and metabolic dysfunction.^17^ In severe acute COVID-19, disruption of gut barrier integrity and increased tight junction permeability lead to the translocation of the gut microbiome into the blood stream, increasing inflammatory responses, leading to the growth of opportunistic microorganisms, and causing dysbiosis and the emergence of LC.^18^ Also, several species of bacteria lost during acute infection are known potential gut immune modulators. It is thus important to recognize the role of the composition of the gut microbiota because this viral infection could cause intestinal inflammation and deteriorate the gut microbiota, which could further exacerbate COVID-19. Noteworthy such distortions in the gut microbiota can lead to LC.^19^

In this context, this study aims to compare the structure and composition of gut microbiota in patients with post-viral long hauler, both with and without excess weight, to understand underlying relationships with adiposity and inflammation, and to identify microbial signatures that could serve as diagnostic or prognostic markers to improve metabolic interactions/outcomes associated to chronic inflammatory status in these patients.

## Results

### Comparison of anthropometric, biochemical, and inflammatory variables in patients with post-viral long hauler according to body mass index status

The stratification of the population according to BMI resulted in 65 participants with a lower than 27.5 kg/m^2^ and 65 with a BMI higher or equal to 27.5 kg/m^2^. The baseline comparisons of anthropometric measurements, body composition, prevalence of metabolic diseases, and quality-of-life data according to BMI status are reported in Table 1. Participants with higher BMI presented significantly higher differences in all anthropometric variables, including blood pressure (Table 1). The presence of comorbidities such as obesity, type 2 diabetes, hypertension, and dyslipidemia were also significantly more prevalent in patients with higher BMI. On the contrary, the results of the lifestyle questionnaire (PCS12 and MCS12), sadness, adherence to the Mediterranean diet and assessment of physical activity did not present differences according to the groups of BMI (Table 1).

**Table 1:** Comparison of anthropometric measurements, body composition and lifestyle factors between patients with post-viral long hauler categorized by status of BMI from METAINFLAMMATION cohort

Variables	Overall (*n* = 130)	Long COVID Low-BMI (*n* = 65)	Long COVID high-BMI (*n* = 65)	*p* value
Age (years)	51 (9.0)	50 (9.8)	52(8.1)	0.16
Sex = Woman (%)	107 (82.3)	56 (86.1)	51 (78.4)	0.78
Weight (Kg)	77.3 (18.3)	64.3 (9.5)	90.4 (15.6)	**<0.001**
BMI (kg/m^2^)	28.5 (5.9)	23.9 (2.6)	33.1 (4.4)	**<0.001**
Waist (cm)	98.2 (15.4)	87.2 (9.3)	109.4 (12.1)	**<0.001**
Skeletal muscle mass (Kg)	46.6 (8.4)	43.4 (7.4)	49.9 (8.1)	**<0.001**
Body fat (%)	35.2 (8.9)	29.2 (6.5)	41.2 (6.7)	**<0.001**
Visceral fat index	9.1 (4.3)	6.3 (2.5)	12.0 (3.9)	**<0.001**
Metabolic age (years)	53 (14)	42 (11)	64 (8)	**<0.001**
SBP (mmHg)	124 (18)	117 (16)	131 (17)	**<0.001**
DBP (mmHg)	76 (11)	73 (11)	81 (10)	**<0.001**
Prevalence of obesity (%)	35 (19)	0 (0.0)	35 (39)	**<0.001**
Prevalence of diabetes mellitus (%)	12 (6)	2 (2)	10 (11)	**0.03**
Prevalence of hypertension (%)	34 (18)	7 (7)	27 (30)	**<0.001**
Prevalence of dyslipidaemia (%)	46 (25)	16 (17)	30 (33)	**0.02**
Sadness (%)	137 (75)	64 (70)	72 (80)	0.18
PCS12	31.7 (9.7)	33.5 (9.9)	29.8 (9.1)	**0.02**
MCS12	36.9 (12.7)	37.7 (12.5)	36.1 (12.9)	0.38
Mediterranean Diet Adherence Questionnaire (0–14)	7.6 (1.9)	7.7 (2.0)	7.6 (1.9)	0.78
SRQ20 (0–20)	10.8 (4.6)	10.3 (4.7)	11.3 (4.6)	0.19
MET (min/week)	1017 (1182)	1198 (1414)	843 (863)	0.07
Physical activity (hour/week)	4.5 (5.2)	5.2 (6.2)	3.9 (3.8)	0.13

Data was presented as mean (standard deviation) and *p* values. The significance threshold was set at *p* < 0.05 ∗, t-test was used to compare the mean of continuous variables and Chi-square (χ2) to compare categorical variables. Lower BMI refers to BMI <27.5 kg/m^2^ and higher BMI refers to BMI >27.5 kg/m^2^. *p* value column is the comparison of variables’ mean between patients with post-viral long hauler and low BMI and long COVID and high BMI using t-test or Mann-Whitney test, according to the distribution of the data. BMI, Body Mass Index; BMR; Metabolic Rate Basal; DBP, Diastolic blood pressure; MCS12, Mental Component Summary; MET, Metabolic Equivalent of Task; PCS12, Physical Component Summary; SBP, Systolic blood pressure; SRQ20, Self-Reporting Questionnaire-20. *p* value lower than 0.05 in bold type.

The baseline comparisons of biochemical determinations depending on BMI are reported in Table 2. Participants with more BMI presented significantly higher values in markers of glucose metabolism such as glucose (*p* < 0.001), HbA1c (*p* < 0.001), HOMA-IR (*p* = 0.01) and insulin (*p* < 0.001). Also, significant differences were found in lipid profile parameters such as HDL-cholesterol (*p* = 0.001) and triglycerides (*p* < 0.001) between groups of BMI. In this line, no significant differences were found in total cholesterol (*p* = 0.12) and LDL-cholesterol (*p* = 0.11). In addition, participants with higher BMI showed significantly higher values of alkaline phosphatase and alanine transaminase (ALT). No significant differences were found between groups of BMI in albumin (*p* = 0.64), hemoglobin (*p* = 0.18), and AST (*p* = 0.27) in this population.

**Table 2:** Comparison of routine metabolic data among patients with post-viral long hauler categorized by BMI status from the METAINFLAMMATION cohort

Variables	Overall (*n* = 130)	Long COVID-low BMI (*n* = 65)	Long COVID- high BMI (*n* = 65)	*p* value
Glucose (mg/dL)	95 (15)	91 (7)	99 (19)	**<0.001**
HbA1c (%)	5.4 (0.5)	5.2 (0.3)	5.51 (0.5)	**<0.001**
Insulin (ÂμUI/ml)	10.7 (11.0)	6.8 (4.8)	14.4 (13.6)	**<0.001**
HOMA-IR	2.6 (4.3)	1.5 (1.2)	3.7 (4.7)	**0.01**
Albumin (g/dL)	4.6 (0.2)	4.5 (0.3)	4.6 (0.2)	0.64
Hemoglobin (g/dL)	14.4 (1.2)	14.3 (1.0)	14.5 (1.2)	0.18
Total cholesterol (mg/dL)	199 (33)	203 (34)	195 (31)	0.12
LDL-cholesterol (U/L)	118.0 (31.1)	121.7 (30.5)	114.4 (31.7)	0.11
HDL-cholesterol (mg/dL)	60 (16)	63 (15)	57 (17)	**0.001**
Triglycerides (mg/dL)	102 (53)	90 (47)	115 (56)	**<0.001**
Alkaline phosphatase (U/L)	72.5 (22.4)	67.0 (18.4)	77.5 (24.1)	**0.005**
AST (U/L)	23.3 (8.5)	22.5 (7.1)	24.2 (9.7)	0.27
ALT (U/L)	25.2 (12.9)	22.3 (10.7)	28.1 (14.3)	**0.001**

Data was presented as mean (standard deviation) and *p* values. The significance threshold was set at *p* < 0.05 ∗, t-test was used to compare the mean of continuous variables and Chi-square (χ2) to compare categorical variables. Lower BMI refers to BMI <27.5 kg/m^2^ and higher BMI refers to BMI >27.5 kg/m^2^. *p* value column is the comparison of variables’ mean between patients with post-viral long hauler and low BMI and long COVID and high BMI using t-test or Mann-Whitney test, according to the distribution of the data. ALT, Alanine Transaminase; AST, Aspartate Aminotransferase; HbA1c, Glycosylated Hemoglobin Test; HDL, High-Density Lipoprotein; LDL, Low Density Lipoproteins. *p* value lower than 0.05 in bold type.

The baseline comparisons of inflammatory markers depending on BMI status are shown in Table 3. Participants with more BMI presented significantly higher differences in neutrophils (*p* = 0.03), monocytes (*p* = 0.009), and lymphocytes (*p* = 0.04) (Table 3). Also, red cell blood distribution width (RDW) (*p* = 0.002) and erythrocyte sedimentation rate (ESR) (*p* = 0.002) resulted in significantly different between groups. In addition, significant differences were found in traditional inflammatory markers such as LDH (*p* < 0.001), C-reactive protein (*p* < 0.001), fibrinogen (*p* < 0.001) and D-dimer (*p* = 0.02), as well as uric acid (*p* < 0.001). However, no significant differences were found in levels of platelets, IL-6, and ferritin, prothrombin activity, activated partial thromboplastin time, and NT-proBNP between groups of comparison.

**Table 3:** Comparison of specific biochemical measurements and inflammatory markers among patients with post viral long-haulers categorized by BMI status from the META INFLAMMATION cohort

Variables	Overall (*n* = 130)	Long COVID-low BMI (*n* = 65)	Long COVID- high BMI (*n* = 65)	*p* value
Leukocytes (10E^3^/mL)	6.1 (1.6)	5.7 (1.6)	6.4 (1.6)	**0.01**
Neutrophiles (10E^3^/mL)	3.5 (1.2)	3.3 (1.2)	3.7 (1.2)	**0.03**
Monocytes (10E^3^/mL)	0.3 (0.1)	0.3 (0.1)	0.4 (0.1)	**0.009**
Lymphocytes (10E^3^/mL)	2.0 (0.6)	1.9 (0.6)	2.1 (0.6)	**0.04**
Neutrophils/Lymphocytes ratio	1.7 (0.9)	1.7 (0.9)	1.8 (0.9)	0.63
Platelets (10E^3^/mL)	262.6 (64.1)	255.7 (67.6)	269.2 (60.4)	0.11
RDW (%)	13.5 (1.9)	13.3 (0.7)	13.6 (2.6)	**0.002**
ESR (mm)	10.4 (6.9)	8.8 (6.2)	11.9 (7.2)	**0.002**
LDH (U/L)	174.6 (30.8)	164.7 (24.5)	184.7 (33.3)	**<0.001**
Uric Acid (mg/dL)	4.9 (1.3)	4.6 (1.2)	5.3 (1.3)	**<0.001**
C-reactive protein (mg/L)	2.6 (3.4)	2.0 (3.0)	3.3 (3.7)	**<0.001**
IL-6 (pg/mL)	3.3 (2.7)	3.2 (2.7)	3.4 (2.7)	0.23
Ferritin (ng/mL)	93.2 (82.5)	88.3 (71.3)	98.1 (93.03)	0.70
Fibrinogen (mg/dL)	341.5 (65.1)	323.6 (67.4)	359.3 (57.8)	**<0.001**
D-dimer (ng/mL)	362.9 (329.6)	345.4 (373.4)	381.9 (280.8)	**0.02**
Prothrombin activity (%)	103.8 (17.6)	105.1 (14.4)	102.6 (20.4)	0.64
Activated Partial Thromboplastin Time (s)	30.5 (3.1)	30.4 (2.8)	30.5 (3.3)	0.71
NT-proBNP (pg/mL)	62.9 (62.0)	65.4 (50.5)	58.9 (70.8)	0.13

Data was presented as mean (standard deviation) and p values. The significance threshold was set at p < 0.05 ∗, t-test was used to compare the mean of continuous variables and Chi-square (χ2) to compare categorical variables. Lower BMI refers to BMI <27.5 kg/m^2^ and higher BMI refers to BMI >27.5 kg/m^2^. P value column is the comparison of variables’ mean between patients with post-viral long hauler and low BMI and long COVID and high BMI using t-test or Mann-Whitney test, according to the distribution of the data. ESR, Erythrocyte Sedimentation Rate; IL-6, Interleukin-6; LDH, Lactate Dehydrogenase; NT-proBNP, Natriuretic Peptide Tests; RDW, Red Cell Blood Distribution Width. P value lower than 0.05 in bold type.

### Analysis of gut microbiota community structure in patients with post-viral long hauler according to body mass index status

**Figure 1:**
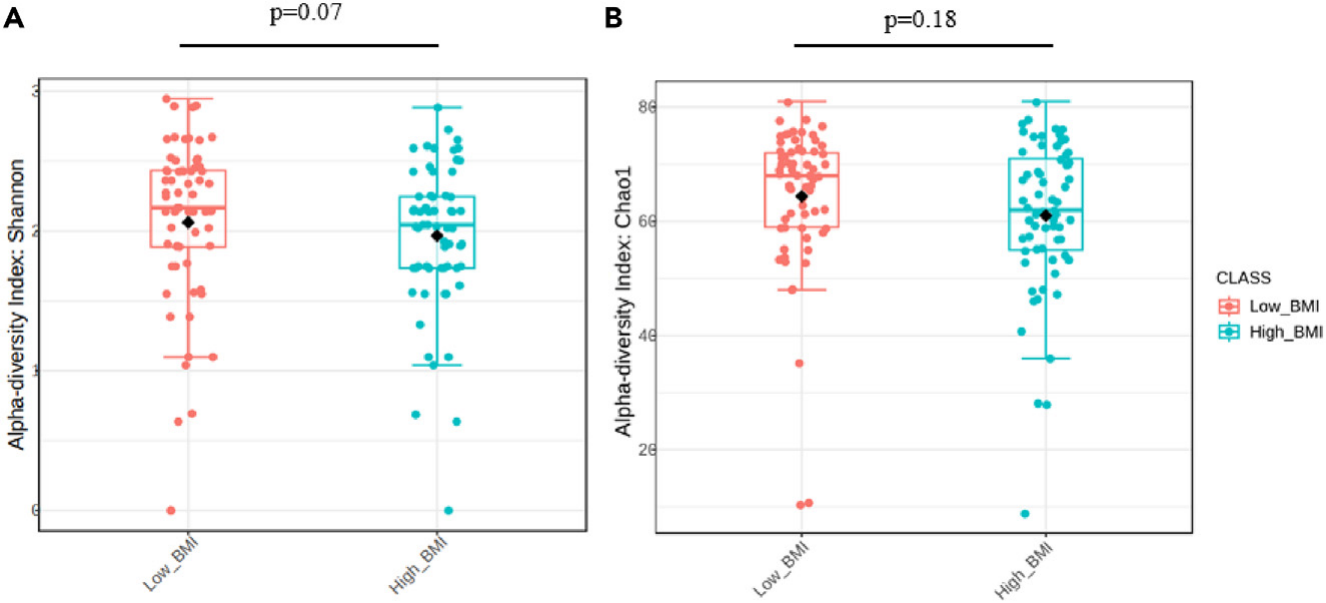
Comparison of community profiling between patients with post-viral long hauler according to BMI status (A) Alpha diversity analysis evaluated by Shannon index. (B) Alpha diversity measured by Chao1 index. Orange boxes represent patients with post-viral long hauler with low BMI and blue boxes represent patients with post-viral long hauler with high BMI.

The examination of gut microbiota richness between participants revealed no statistically significant variances (*p* = 0.52). Similarly, analyses of alpha diversity at the species level showed non-significant differences when assessed using Shannon (*p* = 0.07) and Chao1 (*p* = 0.18) indices (Figures 1A and 1B), but with a trend in Shannon diversity. Likewise, no significant differences in beta diversity were observed using Bray Curtis distances (*p* = 0.48).

**Figure 2:**
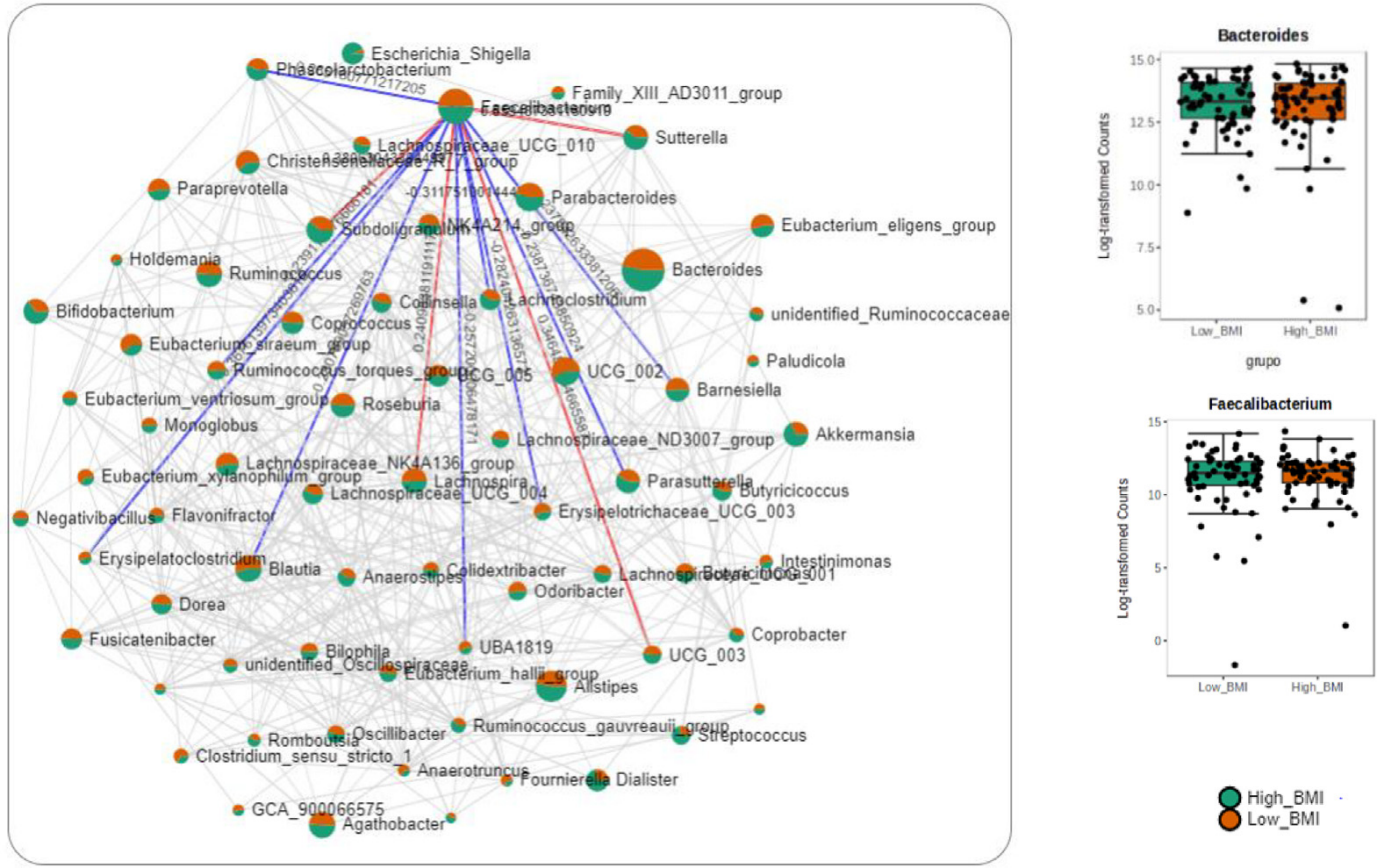
Correlation network performed by SECoM Each node represents a taxon with a color based on the genus level and the size is based on the number of connections to a taxon. Two taxa are connected by an edge if the correlation between the two taxa meets the *p* value (*p* < 0.05) and correlation thresholds (r = 0.3). The blue bars represent negative correlations while the red bars represent positive correlations. The deeper the color of the bar, the stronger the correlation. The boxplot shows the abundances compared by groups of BMI of the selected taxa from the network.

The SECoM analysis was used to assess the network correlations between different bacteria for identifying inherent patterns and correlations. Figure 2 shows the correlation network of the gut microbiota in patients with post-viral long hauler according to BMI. This figure shows that *Bacteroides* and *Faecalibacterium* are the two main genera in the correlation network (Figure 2).

### Analysis of differential abundance and identification of significant microbial features related to inflammatory variables of patients with post-viral long hauler according to body mass index

The linear discriminant analysis effect size (LEfSe) revealed distinctive microbial signatures between participants with autoimmune inflammation according to BMI. Specifically, individuals with high BMI exhibited more abundance of taxa *Candidatus_Soleafarrea* and *Prevotella* genera. Conversely, the gut microbiota composition of the low BMI group was marked by an overrepresentation of taxa such as *Christensenellaceae, Akkermansia, Intestimonas, Paludicola, or Holdemania* (Figure S1).

**Figure 3:**
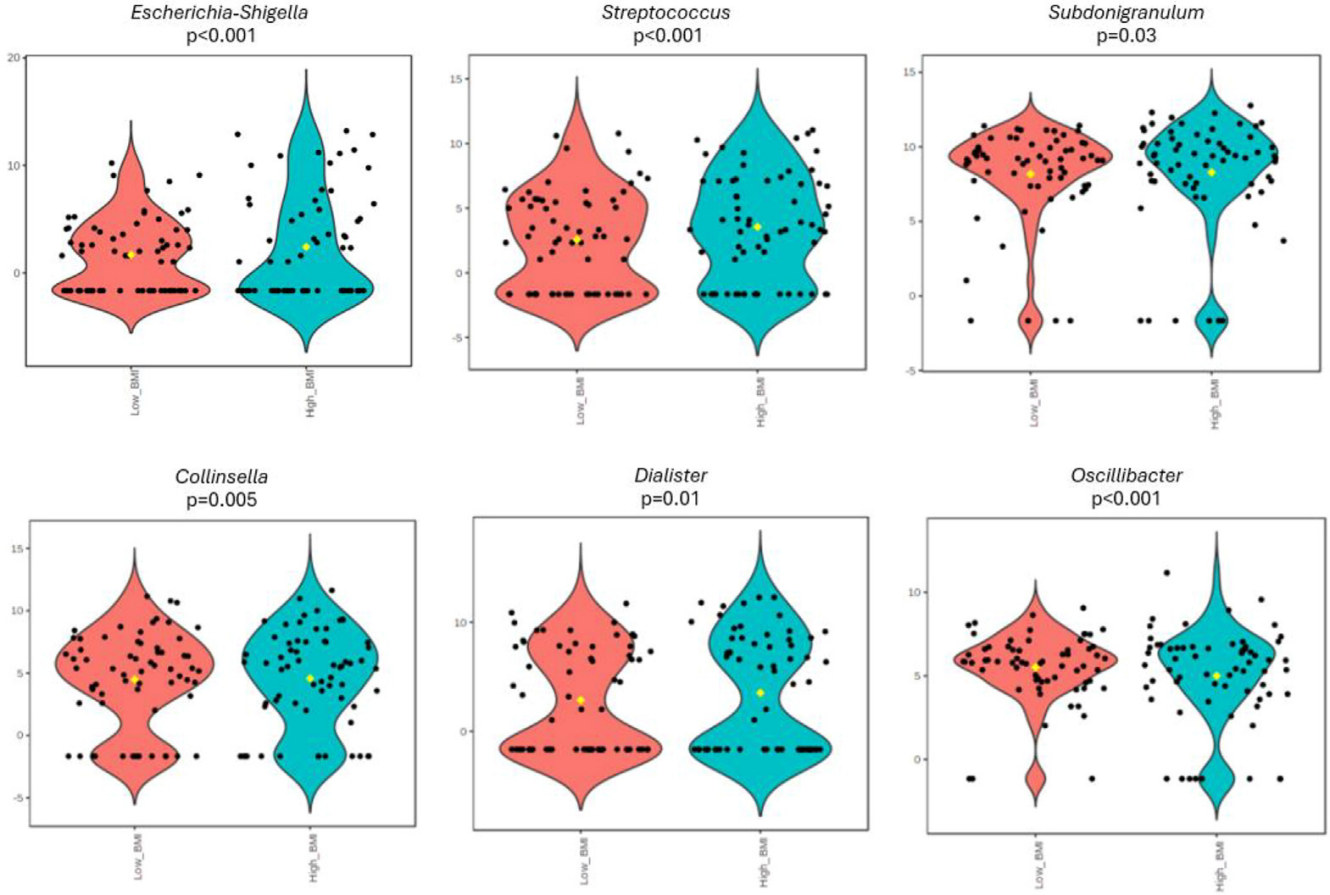
Significant gut microbial changes between patients with post-viral long hauler according to BMI Boxes represent abundance of taxa of low-BMI (red) and high-BMI (blue). Values on Y axis are expressed as log-transformed counts. FDR: false-discovery rate-adjusted *p*-value of EdgeR analysis using Microbiome Analyst.

The analysis of differential abundance between patients with long post-viral long hauler according to BMI status revealed that at genus level, participants with higher BMI presented significantly more abundance of *Escherichia-Shigella* (*p* < 0.001), *Streptococcus* (*p* < 0.001)*, Collinsella* (*p* = 0.005) *Dialister* (*p* = 0.01) and *Subdonigranulum* (*p* = 0.03). Lower abundances were found in participants with high BMI in *Oscillibacter* genus (*p* < 0.001) (Figure 3).

**Figure 4:**
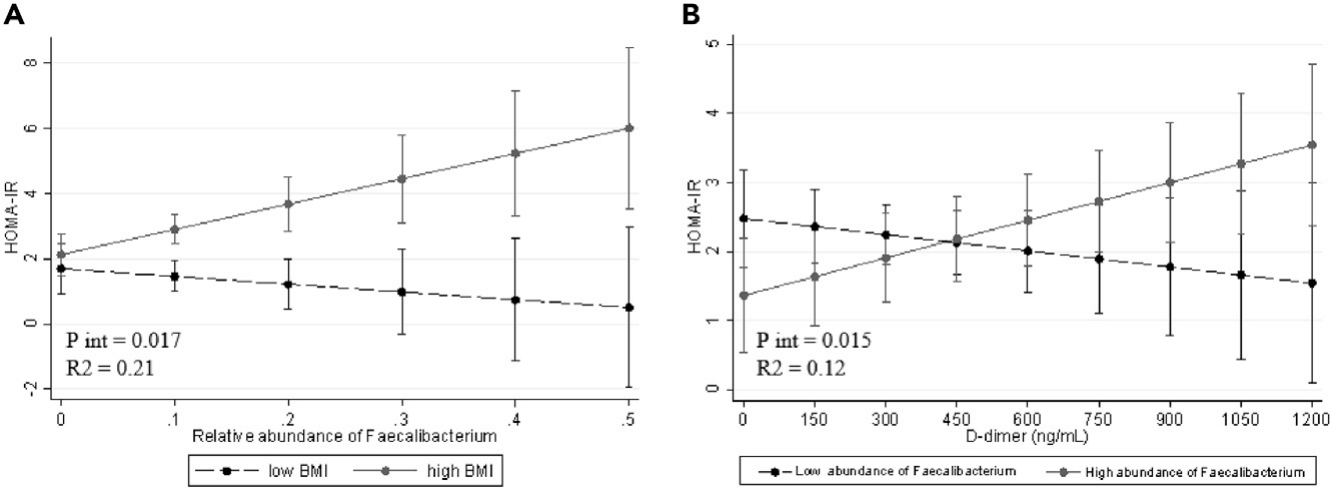
Interactive association of *Faecalibacterium* abundance with HOMA-IR and D-dimer levels in long-haulers stratified by BMI (A) Predicted values of HOMA-IR (dependent variable in the Y axis) according to the relative abundance of *Faecalibacterium* genus (X axis), dashed line represents patients with long-haulers with low-BMI and the solid line represents patients with high BMI. (B) Predicted values of HOMA-IR (dependent variable in Y axis) according to D-dimer levels (X axis). Figure 4B was calculated with <1 (dashed line) and >1 (solid line) relative abundance of *Faecalibacterium*, using linear regression models adjusted for age and sex, showing an association between the relative abundance of *Faecalibacterium*, D-dimer, and insulin resistance measured by HOMA-IR.

Given the differences found in markers of glucose metabolism, a regression model was performed using HOMA-IR as dependent variables and significant and gut microbiota features and BMI as independent variables, including the term of the interaction. A significant interaction was observed between BMI status and *Faecalibacterium* abundance. The Figure 4A shows that participants with high BMI and higher levels of *Faecalibacterium* exhibited elevated HOMA-IR values in this population. In other words, the presence of high levels of *Faecalibacterium* seems to modulate the effects of elevated BMI on insulin resistance, as reflected in HOMA-IR values.

A similar regression model was fitted using HOMA-IR as the dependent variable, and D- Dimer and *Faecalibacterium* abundance as independent variables to explore the relationships with inflammatory outcomes. The Figure 4B shows the significant interaction between D-dimer levels, *Faecalibacterium* abundance, and HOMA-IR in this cohort. The interaction plot illustrates that the patients with elevated D-dimer levels (particularly exceeding 600 ng/mL) and a high abundance of *Faecalibacterium* genus, showed higher predicted values of HOMA-IR, suggesting a possible modulation of insulin resistance depending on D-dimer levels and *Faecalibacterium* abundance.

## Discussion

This investigation aimed to analyze and compare the intestinal microbiota of post-viral patients with persistent symptoms based on body weight measured by BMI, as well as to study potential mechanisms underlying the relationship between intestinal microbiota and inflammatory markers in these populations. The participants of this research featured elevated glucose levels, HbA1c, fasting insulin, triglycerides, and ALT in patients with higher BMI, which emphasizes the association between body weight and metabolic abnormalities.^20^^,^^21^^,^^22^ In addition, patients with post-viral long hauler with higher BMI display hematological patterns marked by increased neutrophil and monocyte counts, as well as elevated levels of lymphocytes.^23^ These observations show a potential link between excess weight and an imbalanced immune response, which may impair the inflammatory status and the consequently tissue injury in individuals with chronic inflammatory conditions.^24^

In addition, the present scientific literature shows that the composition of microbiota may also be linked to the severity of COVID-19 disease. It appeared that patients with COVID-19 with severe disease had more dysbiotic and proinflammatory microbiota, with a higher abundance of opportunistic pathogens such as *Escherichia coli*, *Klebsiella pneumoniae,* and the *Enterococcus* genus, as well as *Streptococcus* spp*,* linked to the expressions of proinflammatory cytokines.^25^ In this line, in this study we showed that *Escherichia-Shigella* and *Streptococcus* genera were more abundant in patients with LC and higher BMI. Moreover, the analysis of the structure of microbiota revealed *Faecalibacterium* and *Bacteroides* as the most significant bacteria among the participants depending on BMI in the gut ecosystem. Some authors have previously found a relationship between *Faecalibacterium* and *Bacteroides* with severe infections of COVID-19.^26^ Indeed, *Bacteroides* genus could downregulate ACE-2 receptor expression, restricting viral replication in the gut.^26^ In this investigation, the regression analysis revealed a relationship between *Faecalibacterium* abundance and D-dimer levels alongside an elevated HOMA-IR index. *Faecalibacterium*, recognized as a key butyrate-producing bacterium within the human gut microbiota, holds key roles in maintaining gut barrier integrity, immunomodulation, and metabolic homeostasis.^27^^,^^28^ Elevated ESR, LDH, uric acid, CRP, IL-6, RDW, fibrinogen, and D-dimer levels in patients with higher BMI reflect a state of heightened immune activation and dysregulated coagulation pathways associated with excess adiposity in the context of LC.^29^^,^^30^ These markers may indicate of ongoing tissue damage, immune cell activation, and alterations in hemostasis, all of which are influenced by the composition and activity of the gut microbiota.^29^

Numerous studies have highlighted the pivotal role of gut microbiota in the pathogenesis of both obesity and type 2 diabetes (T2DM), owing to shared pathological features.^31^ Specifically, alterations in the abundance of Gram-negative bacteria have been implicated in triggering chronic low-grade inflammation characteristic of these metabolic disorders. Furthermore, certain genera, such as *Akkermansia, Faecalibacterium*, and *Bacteroides,* have been inversely associated with obesity and glucose metabolism impairment.^32^ However, notable differences in gut microbiota composition exist between obesity and T2DM. In fact, T2DM is characterized by a *significant reduction in butyrate-producing bacteria, including Faecalibacterium prausnitzii, Roseburia intestinalis*, *Bacteroides intestinalis,* and *Eubacterium rectale*, unlike obesity.^31^^,^^32^^,^^33^

In fact, one of the species lost in COVID-19 associated dysbiosis is *Faecalibacterium prausnitzii* (from *Faecalibacterium* genus), which has been shown to have a role in the inhibition of the production of pro-inflammatory cytokines.^34^^,^^35^^,^^36^ On the other hand, D-dimer, a fibrin degradation product, serves as a biomarker of ongoing fibrinolysis and thrombotic activity. Elevated D-dimer levels observed in patients with post-viral long hauler with higher BMI may reflect a hypercoagulable state and increased thrombotic risk associated with adiposity and chronic inflammation.^37^ Emerging evidence suggests that alterations in gut microbiota can influence coagulation pathways, promoting the translocation of microbial products, such as lipopolysaccharides, into the systemic circulation and triggering immune activation and coagulation cascades.^38^ Furthermore, specific gut microbial taxa, including *Faecalibacterium*, could be implicated in modulating host coagulation processes through the production of bioactive metabolites and interaction with the host immune system.^39^ In this line, *Faecalibacterium* abundance has been inversely correlated with markers of systemic inflammation and coagulation activation, suggesting a relationship in thrombotic events.^39^

The relationship among insulin resistance, D-dimer, and *Faecalibacterium* in patients with chronic inflammation could be explained through several physiological mechanisms.^40^ In fact, insulin resistance can induce the activation of inflammatory pathways such as the nuclear factor kappa B (NF-κB) pathway and the production of proinflammatory cytokines.^41^ This chronic inflammation also contributes to the development of insulin resistance and metabolic dysfunction. Insulin resistance and chronic inflammation can trigger the activation of the coagulation cascade and increase the production of procoagulant factors such as tissue factor and von Willebrand factor, promoting thrombus formation.^42^ Additionally, chronic inflammation can lead to endothelial dysfunction and increased release of D-dimer, a fibrinolytic degradation product, which correlates with coagulation activation. In this context, this investigation identified an interactive relationship between D-dimer levels and *Faecalibacterium* genus abundance, concerning insulin resistance in this population.^43^ These findings suggest that patients with elevated D-dimer levels exceeding 450 mg/dL, despite exhibiting a relatively high abundance of Faecalibacterium, demonstrate higher predicted values of insulin resistance as measured by HOMA-IR. In contrast, individuals with lower D-dimer levels (<450 ng/mL), where higher *Faecalibacterium* abundance were associated with lower HOMA-IR and reduced insulin levels.^44^ At elevated D-dimer levels surpassing 450 ng/mL, the observed absence of *Faecalibacterium*’s protective effect against insulin resistance prompts speculation regarding the interplay between inflammation, coagulation cascades, and gut microbiota modulation.

In this context, it is plausible that heightened D-dimer levels, indicative of increased fibrin degradation and thrombotic propensity, may reflect a prothrombotic state perpetuated by chronic inflammation, characteristic of conditions such as LC. The absence of *Faecalibacterium*’s protective influence in individuals with elevated D-dimer levels may hint at potential disruptions in gut microbiota-immune interactions. *Faecalibacterium* may exert its beneficial effects through various mechanisms, including SCFA production, regulation of immune responses, and modulation of gut barrier integrity.^45^ However, in the presence of higher levels of D-dimer which signify elevated thrombotic and inflammatory states, the immunomodulatory capacity of *Faecalibacterium* could be compromised, as a possible explanation. Another plausible mechanism involves alterations in gut barrier integrity and mucosal permeability. *Faecalibacterium* has been associated with the maintenance of gut barrier function in the scientific bibliography, thereby preventing the translocation of microbial products and inflammatory mediators into the systemic circulation.^46^^,^^47^ However, in conditions characterized by elevated D-dimer levels and systemic inflammation, gut barrier integrity may be compromised, leading to increased mucosal permeability and microbial translocation. This phenomenon can trigger systemic immune activation and metabolic disturbances, exacerbating insulin resistance despite the presence of *Faecalibacterium*.^48^ Besides, *Faecalibacterium*-derived metabolites, such as SCFAs mentioned above, play a crucial role in adipose tissue function and insulin signaling pathways. Nonetheless, under conditions of heightened inflammation and coagulation represented by elevated D-dimer levels, alterations in SCFA production and adipose tissue metabolism may occur, impairing insulin sensitivity despite the presence of *Faecalibacterium*.^49^^,^^50^^,^^51^ These potential mechanisms are speculative explanations for the observed interactive association in this population; however, they could not be confirmed, thus serving as part of the study’s limitations. For instance, the determination of SCFA in these patients could have offered further insights.

This investigation presents some limitations. Firstly, the retrospective nature of the study design limits causal inference, and longitudinal studies are needed to establish temporal relationships between variables. Secondly, the relatively small sample size and homogeneity of the study population may limit the generalizability of the findings to broader populations, and errors I and error II cannot be ruled out. Also, this investigation needs further research to be generalizable across diverse populations. Despite these limitations, this study provides valuable insights into the complex interplay between metabolic and inflammatory conditions in viral long haulers depending on BMI and underscores the need for further research to elucidate underlying mechanisms and develop targeted interventions. The study’s focus on investigating the role of *Faecalibacterium* in modulating insulin resistance and coagulation within the context of LC represents a novel and highly relevant contribution, especially considering the ongoing global health challenges posed by COVID-19 and its long-term effects. Moreover, the utilization of comprehensive methodologies, such as 16S rRNA sequencing for microbiota analysis and a range of biochemical markers for metabolic health assessment, enhances the robustness and reliability of the study’s findings. Furthermore, the insights gleaned from this research regarding the interactive relationship between gut microbiota, particularly *Faecalibacterium*, and metabolic health markers in patients with long COVID hold the potential for targeted therapeutic interventions, thereby offering significant clinical impact.

### Conclusions

This research evidence that anthropometric, biochemical variables related to glucose metabolism such as insulin resistance and inflammatory markers such as D-dimer are increased in patients with post-viral long haulers with higher BMI. Furthermore, these findings showed an effect modification on HOMA-IR levels involving the genus *Faecalibacterium* and D-dimer levels, highlighting the complexity of interactions between the intestinal microbiota and the host. Considering adiposity and intestinal microbiota in therapeutic approaches may be the precise clinical management of patients with post-viral long haulers.

## STAR★Methods

### Key resources table

**Table:** 

REAGENT or RESOURCE	SOURCE	IDENTIFIER
**Critical commercial assays**		

DNA Kit for feces		
Automated hematology analyzer	Roche	SYSMEX XN-20
Autoanalyzer (Atellica™ Solution)	-	-
ELISA kit for C-reactive protein (CRP)	Sigma-Aldrich	Cat# RAB0096
ELISA kit for fibrinogen	Sigma-Aldrich	Cat# RAB0146
ELISA kit for insulin	Sigma-Aldrich	Cat# RAB0329
ELISA kit for lactate dehydrogenase (LDH)	Sigma-Aldrich	Cat# MAK066
ELISA kit for D-dimer	Sigma-Aldrich	Cat# RAB0532
ELISA kit for NT-proBNP	Sigma-Aldrich	Cat# RAB0532
ELISA kit for interleukin-6 (IL-6)	Sigma-Aldrich	Cat# RAB0306
ELISA kit for prothrombin activity	Sigma-Aldrich	Cat# RAB0135
OMNIgene® •GUT kits	DNA Genotek	
QIAamp® DNA kit	Qiagen	Cat# 51504
MiSeq System	Illumina	
PCR primers	-	16S Amplicon PCR Forward Primer: 5′ TCGTCGGCAGCGT CAGATGTGTATAAGAGACAGCCTACGGGNGGCWGCAG 16S Amplicon PCR Reverse Primer: 5′ GTCTCGTGGGCTCGGA GATGTGTATAAGAGACAGGACTACHVGGGTATCT AATCC
Nextera XT DNA Library Preparation Kit	Illumina	Cat# FC-131-1024

**Software and algorithms**		

R-studio	CRAN	Version 4.2
QIIME2	Bolyen et al., 2019	https://docs.qiime2.org/2020.2/install/
DADA2		
SILVA v.132 database		
Phyloseq	McMurdie and Holmes, 2013	http://www.bioconductor.org/packages/release/bioc/html/phyloseq.html
MicrobiomeAnalyst		https://www.microbiomeanalyst.ca/
Stata	StataCorp LLC	http://www.stata.com

**Other (Anthropometric measurements and quality of life data)**		

Bioimpedance scale (TANITA SC-330)	Tanita Corporation	Cat# TANITA SC-330
Standard tape measure	-	-
SF-12 questionnaire	Quality Metric Incorporated	
IPAQ questionnaire	International Physical Activity Questionnaire	https://sites.google.com/site/theipaq/
MEDAS-14	PREDIMED Study	https://predimed.onmedic.net
FFQ (20 items) questionnaire	University of Barcelona	

### Resource availability

#### Lead contact

Further information and requests for resources, data availability and reagents should be directed to and will be fulfilled by the lead contact, Amanda Cuevas-Sierra (amanda.cuevas@alimentacion.imdea.org).

### Experimental model and Study participant details

#### Study design

This investigation was performed on 130 adult individuals from the METAINFLAMMATION project (ref. Y2020/BIO-6600), using baseline data. Participants were recruited between January 2022 and June 2023 at the Internal Medicine Service of Puerta de Hierro Majadahonda University Hospital (Madrid, Spain). Inclusion in the study was contingent upon participant acceptance and signing of the informed consent form. The study adhered to the principles outlined in the Declaration of Helsinki and received approval from the Research Ethics Committee of Puerta de Hierro Majadahonda University Hospital (File Number PI 164-21). All data collection procedures followed approved ethical guidelines and validated hospital protocols.

#### Participants

This research involved 130 viral long-haulers adults, with Caucasian and Hispanic ancestry, with putative pathophysiological inflammation underlying a viral infection and followed the guidelines of the National Institute for Health, and Care Excellence (NICE) and National Institute for Health and Care Research (NIHR).^52^^,^^53^ Participants met the following inclusion criteria: age >18 years, BMI >17.01 kg/m^2^ and <51.35 kg/m^2^ and a diagnosis of long-COVID confirmed by the medical staff of the Internal Medicine service of the Puerta de Hierro Majadahonda University Hospital (Madrid, Spain). In addition, they provided fecal samples, which were sequenced by a specialised external laboratory. The exclusion criteria included the presence of severe psychiatric disorders, the use of weight-modifying agents, pregnancy, or lactation and the consumption of probiotics, antibiotics or any medication known to alter the composition of the intestinal microbiota at least 3 weeks before the fecal sample collection, as well as the rejection to participate.

### Method details; quantification and statistical analysis; additional resources

#### Anthropometrics measurements

Anthropometric measurements, including body weight and height, plus waist circumference, along with body composition as determined by bioimpedance, were obtained by a skilled dietitian and clinical staff using appropriate equipment and validated methods. Body weight was determined using a bioimpedance scale (TANITA SC-330; Tanita Corporation), which also provided estimates of body composition. Waist and hip circumferences were measured with a standard tape measure following established protocols and performed by trained dietitians.^54^ Body mass index (BMI) was calculated as the ratio of body weight to the square of height (kg/m^2^), and the international WHO criteria were applied (BMI within normal weight <24.9 kg/m^2^; BMI within overweight <29.9 kg/m^2^; BMI in obesity ≥30 kg/m^2^).^55^ Metabolic age was assessed based on sex, body composition and metabolic rate data (TANITA SC-330; Tanita Corporation).^56^

#### Biochemical data

Blood samples were obtained under fasting conditions through venipuncture. These samples underwent analysis for leukocytes, lymphocytes, neutrophils, monocytes, hemoglobin, mean corpuscular volume, platelets, mean corpuscular hemoglobin concentration (CHCM), erythrocyte sedimentation rate (ESR) and erythrocyte distribution width (RDW) utilising an SYSMEX XN-20 automated haematology analyser (Roche, Basel, Switzerland).^53^ The neutrophil/lymphocyte ratio was calculated directly from the measured values. Routine biochemical markers, including glucose, total cholesterol, glycated hemoglobin, alkaline phosphatase, uric acid, ferritin, high-density lipoprotein (HDL), triglycerides, alanine aminotransferase (ALT) and aspartate aminotransferase (AST) were measured following standardised hospital protocols using a quality-controlled autoanalyzer (Atellica Solution) as per established criteria. Prognosis-related variables, proinflammatory factors, and markers such as C reactive protein (CRP), fibrinogen, insulin, lactate dehydrogenase (LDH), D-dimer, N-terminal pro-brain natriuretic peptide type B (NT ProBNP), interleukin-6 (IL-6), and prothrombin activity also followed standardised procedures (using duplicates), primarily employing ELISA kits (Sigma-Aldrich ELISA Kit) as outlined by the suppliers.^57^

#### Clinical measurements and quality of life data

Systolic and diastolic blood pressures were measured using a sphygmomanometer, adhering to standardised criteria in accordance with international guidelines.^58^ Patients completed several validated questionnaires covering sociodemographic information, family history, health-related quality of life (assessed with the 12-item Short Form Survey, SF-12) and lifestyle factors such as physical activity (evaluated using the International Physical Activity Questionnaire, IPAQ) and dietary patterns (assessed using the 14-point Mediterranean Diet Adherence Screener, MEDAS-14 and a validated short FFQ with 20 items), under the guidance of a trained dietitian.^59^^,^^60^^,^^61^^,^^62^

#### Metagenomic analysis

Fecal samples were self-collected by the volunteers using OMNIgene •GUT kits (DNA Genotek, Ottawa, ON, Canada), according to the supplier instructions. Samples were aliquoted in 2mL tubes for the storage at −80°C (by duplicate). Bacterial DNA was isolated with the QIAamp DNA kit (Qiagen, Hilden, Germany) following the manufacturer’s protocol. The V3-V4 hypervariable regions of the 16 S rRNA gene were amplified by paired-end DNA sequencing in the MiSeq System (Illumina, San Diego, CA, USA) at Novogene Sequencing- Europe (Cambridge, United Kingdom). The primers used for the PCR reactions were (16S Amplicon PCR Forward Primer = 5 0 TCGTCGGCAGCGTCAGATGTGTATAAGAGACAGCCTACGGGNGGCWGCAG; 16S Amplicon PCR Reverse Primer = 5 0 GTCTCGTGGGCTCGGAGATGTGTATAAGAGACAGGACTACHVGGGTATCT AATCC).

Amplicon preparation was performed by using the 16S Metagenomic Sequencing Library Preparation Protocol (Illumina, San Diego, CA, USA). This protocol also includes overhang adapter sequences for compatibility with Illumina index and sequencing adapters. Nutrients 2021, 13, 1738 4 of 13 Amplicon size was subsequently verified by electrophoresis (LabChip GX; PerkinElmer, Waltham, MA, USA). DNA libraries for 16S rRNA amplicon sequencing were prepared using the Nextera XT DNA Library Preparation Kit (Nextera XT) (Illumina, San Diego, CA, USA) according to the manufacturer’s instructions. The 16S rRNA libraries were sequenced with the Illumina MiSeq benchtop sequencer in paired-end mode with 2 × 300 cycles using the MiSeq Reagent v3 600-cycle kit (Illumina, San Diego, CA, USA). The low-quality reads were filtered, and chimeric sequences were removed after alignment using the Quantitative Insights into Microbial Ecology program (QIIME2).^63^^,^^64^ The subsequent clean reads were clustered as amplicon sequence variants (ASVs) using DADA2 and annotated with the SILVA v.132 16S rRNA gene database. Relative abundance of each ASV was calculated using the Phyloseq R package. MicrobiomeAnalyst web-based platform (https://www.microbiomeanalyst.ca/) was used for analysis of alpha and beta diversity, using Shannon and Chao1 indexes and using Bray-Curtis distances represented using principal coordinates analysis (PCoA) and PERMANOVA test, respectively. Data were filtered by a minimum count of 4, 20% of prevalence in samples, and a low variance filter (maximum percentage of samples to remove) of 20%, based on inter-quantile range. Data were then normalized by the centered-log ratio (CLR).

#### Statistical analyses

Participants were stratified according to median BMI, being "lower BMI" (for those with values below 27.5 kg/m^2^) or "higher BMI" (for those with values above or equal 27.5 kg/m^2^) for convenient statistical analyses. Variables were expressed as means and standard deviations for quantitative variables and number of cases and percentage for qualitative variables. Student’s t tests were implemented to compare the means of the continuous variables at the beginning of the study and the categorical variables were statistically analyzed using the chi-square (χ2) test. The SECoM (Significance-Edge-Consistency Method) process were implemented for identifying significant correlations among the abundances of different bacteria in microbiome samples (correlation threshold established = 0.3). This analysis utilizes a network-based approach to visualize and analyze these correlations, allowing for the identification of the most relevant and consistent microbial interactions within the dataset. The LEfSe analysis was performed using MicrobiomeAnalsyst platform and using a *p*-value cutoff of 0.05 FDR-adjusted and log LDA score = 2.0. EdgeR was used for assessed the analysis of differential abundance of bacterial taxa between groups, corrected by FDR. Potential interactions were investigated with general linear regression models that introduced the corresponding interaction terms into the models, which were adjusted for age and sex and using Stata 12. (StataCorp LLC, College Station, TX, USA; http://www.stata.com). A *p* value of <0.05 was considered statistically significant. The normalization CLR was used for the regression analysis, as recommended.^65^^,^^66^
