# Supplementary material for: Modulatory role of Faecalibacterium on insulin resistance and coagulation in patients with post-viral long haulers depending on adiposity
Source: iScience. 2024 Jul 6;27(8):110450. doi: 10.1016/j.isci.2024.110450 (PMC11284562; doi:10.1016/j.isci.2024.110450)
Supplement: Document S1. Figure S1 [file mmc1.pdf]

iScience, Volume *27*

Supplemental information

Modulatory role of *Faecalibacterium* on insulin resistance and coagulation in patients with post-viral long haulers depending on adiposity

Amanda Cuevas-Sierra, Lourdes Chero-Sandoval, Andrea Higuera-Gómez, J. Antonio Vargas, María Martínez-Urbistondo, Raquel Castejón, and J. Alfredo Martínez

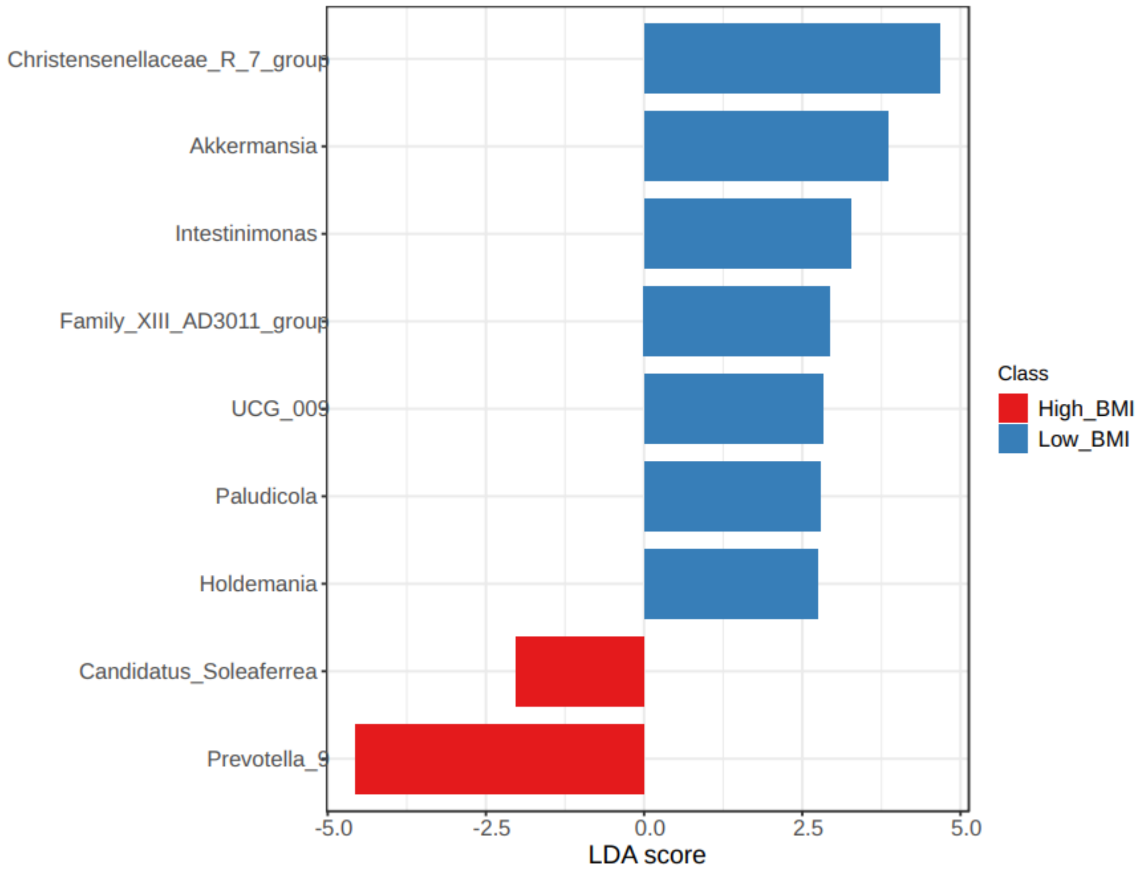

Figure S1. Linear discriminant analysis between groups of BMI. The most differentially abundant taxa between low and high BMI are represented in a bar graph according to the LDA score (log 10), an estimation of the effect size. Only taxa meeting a $P<0.05$ and LDA score significant threshold |>2| are shown. Red, bacterial taxa statistically overrepresented in patients with autoimmune inflammation and high BMI; blue, bacterial taxa overrepresented in participants with autoimmune inflammation and low BMI.
